# Somatic *PRKAR1A* mutation in sporadic atrial myxoma with cerebral parenchymal metastases: a case report

**DOI:** 10.1186/s13256-019-2317-z

**Published:** 2019-12-25

**Authors:** Ashley Roque, Tara Kimbrough, Christopher Traner, Joachim M. Baehring, Anita Huttner, Jennifer Adams, Sandra Canosa, Jeffrey Sklar, Joseph A. Madri

**Affiliations:** 1 Departments of Neurosurgery, Yale University School of Medicine, New Haven, CT USA; 2 Departments of Radiology, Yale University School of Medicine, New Haven, CT USA; 3 Departments of Pathology, Yale University School of Medicine, 310 Cedar Street, P.O. Box 208023, New Haven, CT 06520-8023 USA

**Keywords:** Sporadic cardiac myxoma, Brain metastases, *PRKAR1A* mutation

## Abstract

**Background:**

Atrial myxomas are generally considered benign neoplasms. The majority of tumors are sporadic and less than 10% are associated with an autosomal dominant condition known as the Carney complex, which is most often caused by germline mutation in the gene *PRKAR1A*. Whether this gene plays a role in the development of sporadic myxomas has been an area of debate, although recent studies have suggested that some fraction of sporadic tumors also carry mutations in *PRKARIA*. Extra-cardiac complications of atrial myxoma include dissemination of tumor to the brain; however, the dissemination of viable invasive tumor cells is exceedingly rare.

**Case presentation:**

We present here a 48-year-old white woman who developed multiple intracranial hemorrhagic lesions secondary to tumor embolism that progressed to ‘false’ aneurysm formation and invasion through the vascular wall into brain parenchyma 7 months after resection of an atrial myxoma. Whole exome sequencing of her tumor revealed multiple mutations in *PRKAR1A* not found in her germline deoxyribonucleic acid (DNA), suggesting that the myxoma in this patient was sporadic*.*

**Conclusions:**

Our patient illustrates that mutations in *PRKAR1A* may be found in sporadic lesions. Whether the presence of this mutation affects the clinical behavior of sporadic tumors and increases risk for metastasis is not clear. Regardless, the protein kinase A pathway which is regulated by *PRKAR1A* represents a possible target for treatment in patients with metastatic cardiac myxomas harboring mutations in the *PRKARIA* gene.

## Background

Primary intracardiac neoplasms are rare tumors, with an estimated prevalence of between 0.02 and 0.25% of the population [1–3]. Atrial myxomas originating in the left atrium make up most of these tumors [4, 5]. Greater than 90% of atrial myxomas are sporadic and the rest are the result of a hereditary condition known as the Carney complex. Carney complex is inherited in an autosomal dominant fashion due in most cases to inactivating mutations of the *PRKAR1A* gene and is characterized by pigmented lesions of the skin, myxomas (cardiac and cutaneous), and multiple endocrine tumors [6]. Mutations in *PRKAR1A* were previously not thought to be responsible for the development of isolated, sporadic cardiac myxomas, but genetic alterations in this gene have recently been identified in a minority of such tumor samples [7, 8]. Cardiac myxomas are considered to be benign and cured by complete surgical resection of the cardiac lesion. Recurrences have been observed, but are much more likely to be seen in cases of familial myxoma (12–22% recurrence rate) than sporadic myxoma (1–3%) [4, 9].

Despite their generally benign nature, cardiac myxomas may have devastating consequences due to their location and ability to spread through the blood. Embolic events occur in 30 to 40% of patients with cardiac myxomas and the central nervous system (CNS) is the most frequent site of embolism [4, 10, 11]. This generally manifests as ischemic events, but aneurysmal dilation due to tumor invasion into cerebral vessel walls and resultant intracerebral hemorrhages are also seen [12]. In very rare cases, metastatic disease with clear-cut invasion into the CNS parenchyma is observed. The mechanism behind viable, invasive tumor cell dissemination to the CNS from tumors with benign histopathology is not well understood. There is currently no evidence to the best of our knowledge that patients with tumors due to Carney complex are more likely to experience metastatic consequences. Due to the very small number of patients experiencing CNS metastases, there is no standardized management when they do occur.

Here we describe a rare case of a patient who developed progressive brain metastases as a delayed consequence of tumor embolism a year after removal of an atrial myxoma. Whole exome sequencing of tumor tissue from her heart and brain revealed multiple somatic mutations in *PRKAR1A*. Additional tissue from an earlier benign cystadenoma and buccal swab showed no similar mutation. Our patient had no known family history of myxomas or Carney complex.

## Case presentation

### Clinical course

**Fig. 1:**
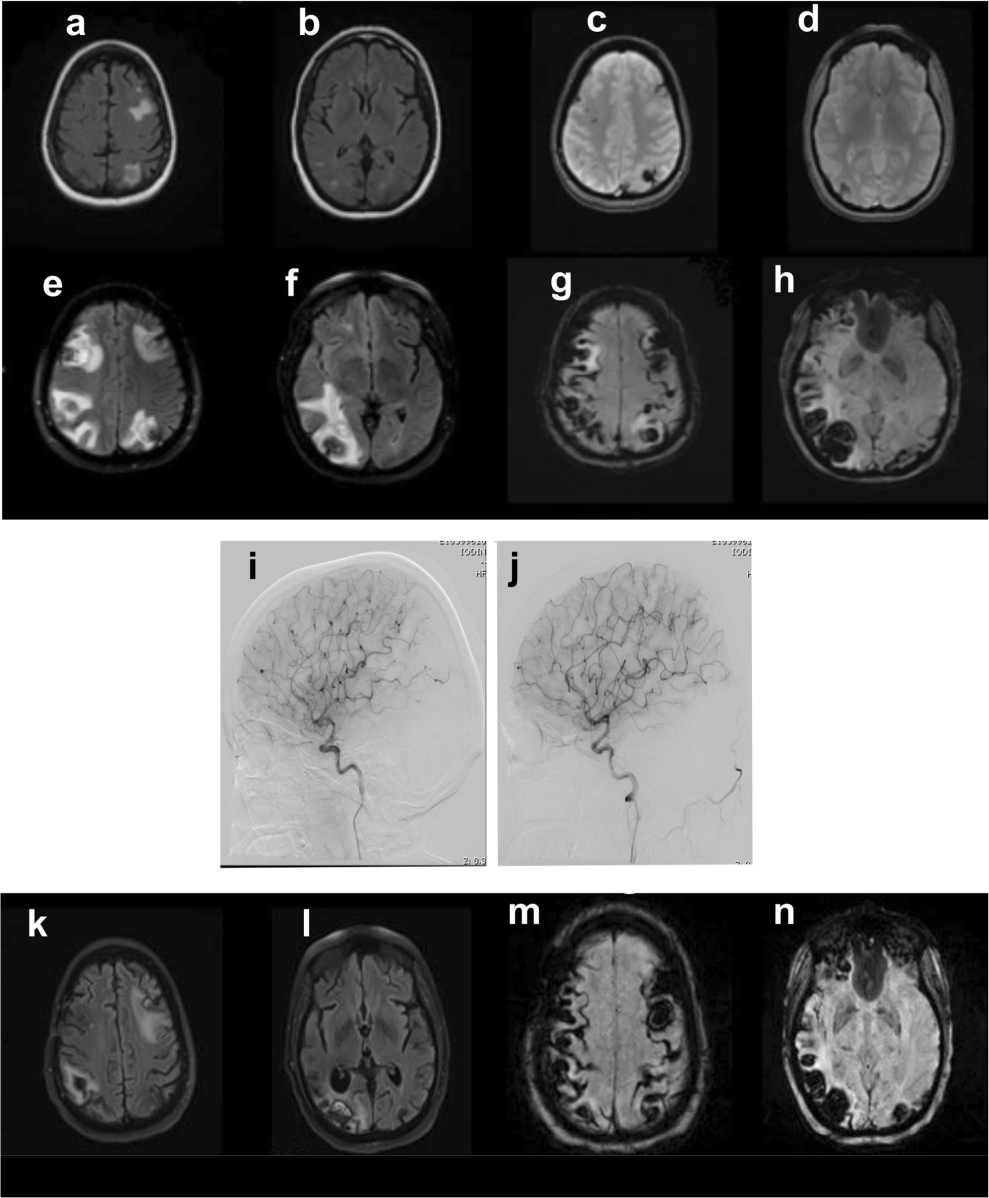
**a**–**n** Timeline of patient: representative images. Representative images from April 2017 (7 months after resection of atrial myxoma) with fluid-attenuated inversion recovery (**a**, **b**) and gradient recalled echo sequences (**c**, **d**) showing small hemorrhagic lesions throughout the bilateral frontal, parietal, and occipital lobes. Follow-up imaging from October 2017 shows interval increase in size of numerous lesions on fluid-attenuated inversion recovery (**e**, **f**) and susceptibility weighted imaging (**g**, **h**). **i** (right internal carotid artery) and **j** (left internal carotid artery) are representative images from cerebral angiogram done in October 2017, demonstrating multiple mycotic aneurysms. Fluid-attenuated inversion recovery (**k**, **l**) and susceptibility weighted imaging (**m**, **n**) sequences from repeat magnetic resonance imaging of the brain in March 2018 (post-radiation)

Our patient is a 48-year-old white woman with a previous history of multiple ovarian cystadenomas requiring a total hysterectomy and bilateral oophorectomy. Her initial neurological symptoms started in April 2016, when she developed daily headaches. Magnetic resonance imaging (MRI) done at an outside institution showed small scattered fluid-attenuated inversion recovery (FLAIR) hyperintensities and areas of susceptibility weighted imaging (SWI) signal intensity in her bilateral occipital lobes, right frontal lobe, and left parietal lobe (see Fig. 1a–n). They were believed at the time to be a consequence of prior trauma. In September 2016, she reported continued headaches and significant fatigue, which prompted workup with a transthoracic echocardiogram (TTE). This revealed a 3.5 × 2.5 × 2.5 cm mass within the left atrium which was felt to most likely represent a myxoma. She underwent successful resection of the lesion shortly thereafter (at another institution), with the outside pathology report confirming an atrial myxoma. Postoperative TTE showed no concern for residual disease. She was well until April 2017, when she experienced recurrent headaches that were now associated with new symptoms of fingertip numbness. A repeat MRI was performed (Fig. 1) and revealed multiple small, enhancing hemorrhagic lesions throughout her bilateral parietal, frontal, and occipital lobes. She then underwent conventional angiography which revealed multiple “mycotic-like” aneurysms in the right anterior and middle cerebral arterial distributions and left middle and posterior cerebral arterial distributions. An infection workup and repeat TTE were both negative. The lesions were followed with serial imaging; 6 months later in October 2017, she experienced an acute event with severe headache, left-sided visual field changes, and severe dizziness while driving, necessitating calling for emergency assistance. She was then transferred to our institution via helicopter for continued management.

An examination at the time of transfer was remarkable for short-term memory impairment as well as an incomplete homonymous hemianopia to the left. There were no appreciable skin lesions. Repeat imaging showed significant increase in size of the previously multiple enhancing hemorrhagic lesions with surrounding edema (Fig. 1). A systemic workup revealed no evidence of additional lesions or recurrent cardiac tumor: computed tomography (CT) of her chest, abdomen, and pelvis; transesophageal echocardiography (TEE); and CT cardiac angiography.

**Fig. 2:**
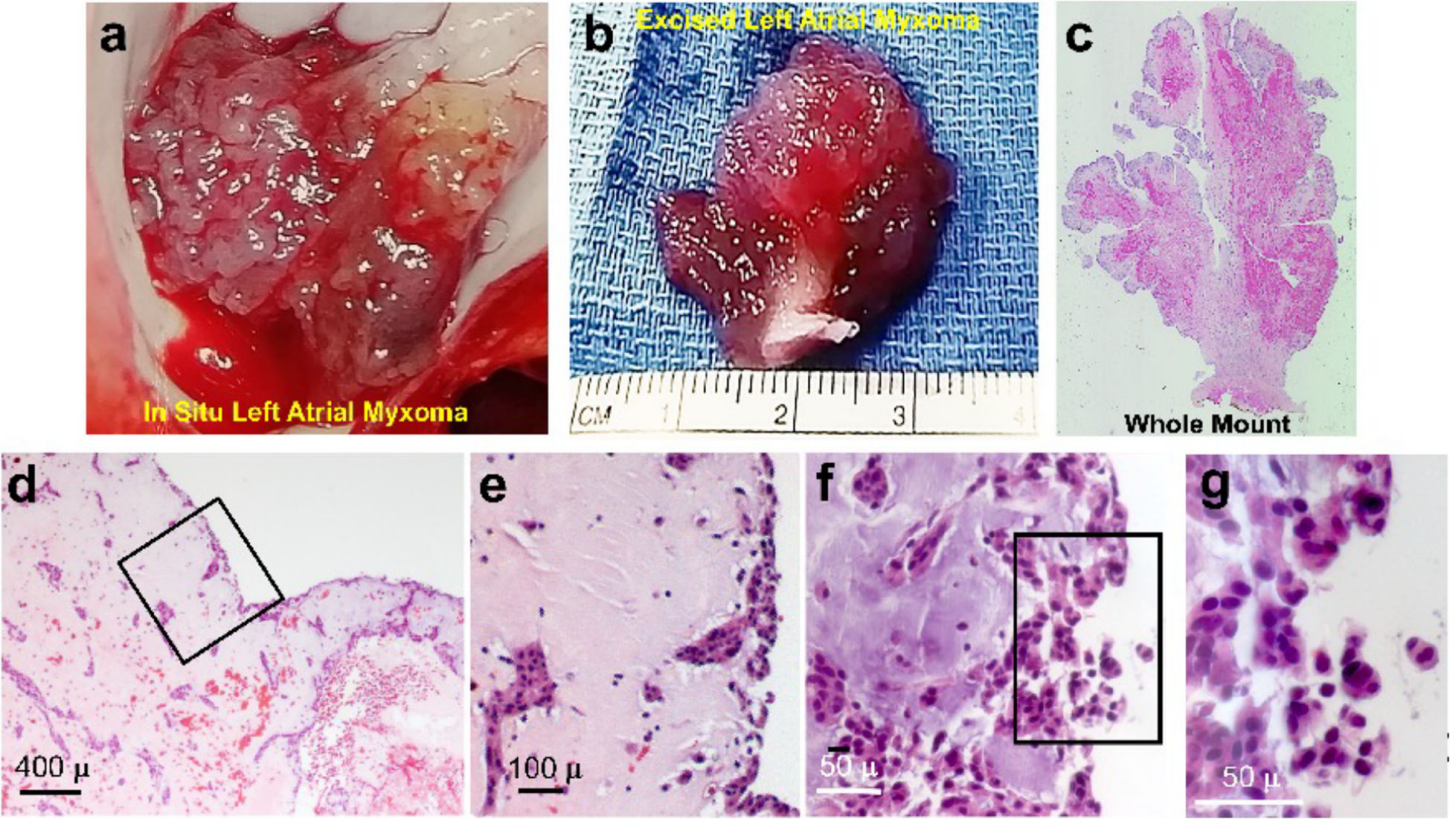
**a**–**g** Left atrial myxoma. **a** *In situ* photograph of the myxoma at the time of surgery consisting of a complex papillary structure comprising grape-like clusters organized into an arborizing network. **b** Photograph of the excised atrial myxoma consisting of a tree-like structure with several arborizing branches. **c** Six micron section of the myxoma whole mount illustrating the arborizing network of grape-like clusters converging on a fibrous stalk. **d, e** Low (**d**) and high (**e**) power images illustrating lepidic cells lining the surfaces (**d**) and cells in a myxoid matrix forming abortive clusters of vessel-like structures (**e**). **f, g** Intermediate (**f**) and high (**g**) power images of occasional clusters of rounded cells at and near the surfaces displaying a lack of cohesion, forming small groups of cells separating from the surfaces of the myxoma

**Fig. 3:**
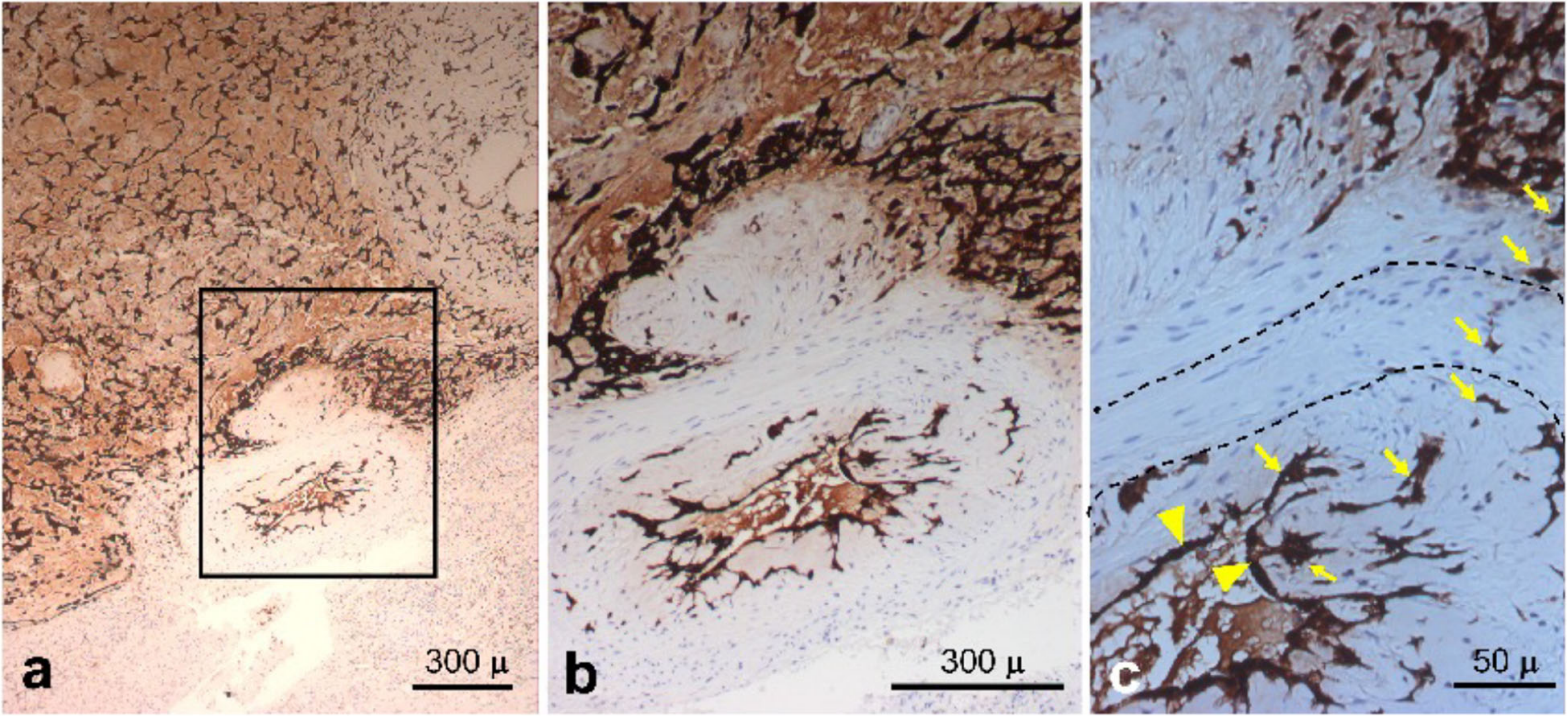
**a**–**c** Micrographs of the image-directed brain biopsy. Micrographs of low (**a**), intermediate (**b**), and high (**c**) power illustrating images of the brain biopsy stained with anti-calretinin antibody. **a** Low power overview of an arterial vessel illustrating replacement of the normal lining endothelium with metastatic myxoma cells stained for calretinin and direct extension of the tumor cells through the vessel wall and into surrounding brain parenchyma. The box denotes the microscopic field presented in panel **b**. **b** Intermediate power image illustrating the replacement of normal endothelium with anti-calretinin-staining atrial myxoma cells and invasion of the vessel wall and brain parenchyma by the myxoma cells. **c** High power image illustrating individual calretinin-positive atrial myxoma cells which have replaced the normal vessel endothelium and the subendothelium (yellow arrowheads) with myxoid matrix and invasion by individual tumor cells through the media of the vessel wall and into adjacent brain parenchyma (yellow arrows). The dashed lines denote the internal and external elastic lamina marking the vascular smooth muscle and extracellular matrix the comprises the media of the vessel

A repeat cerebral angiogram was performed and again showed multiple “mycotic-like” aneurysms, a few of which had decreased in size since the prior examination. Given the unusual nature of her case, it was decided to obtain a brain biopsy to guide treatment. Examination of the histologic sections of the resected myxoma and recuts of the paraffin blocks requested from the outside institution confirmed the diagnosis and revealed clusters of rounded hyperchromatic cells displaying a lack of cohesion, forming small collections of cells separating from the surface of the myxoma (Fig. 2a–g). A biopsy of a right frontal lobe lesion confirmed suspicion of cerebral metastasis from the myxoma and myxomatous infiltration of cerebral blood vessels resulting in pseudoaneurysm formation and tumor cell migration into the parenchyma (Fig. 3; see “Histopathologic analysis” section for detailed description). Based on available reports in the literature and the rapid enlargement of her CNS lesions, she underwent whole brain radiation with hippocampal sparing (3750 cGy in 15 fractions). In light of recent studies, 6 months of memantine was administered to facilitate the prevention of cognitive dysfunction following whole brain radiotherapy [13, 14]. An MRI performed 3 months post radiation (March 2017) showed decrease size of dominant occipital lesions and stability of the remaining lesions.

The lesions have remained stable on the most recent MRIs: December 2018 (1 year post treatment) and June 2019 (18 months post treatment). At the present time, our patient is engaged in cognitive, ocular, and physical therapy to ameliorate the disabilities caused by the cerebral metastases and her subsequent treatments. Whole exome analysis was then performed on tissue from cardiac myxoma, brain metastasis, and ovarian cystadenoma revealing somatic mutations in *PRKAR1A* within the cardiac tumor and brain metastasis, but not in the tissue from the ovarian cystadenomas.

### Histopathologic analysis

Cardiac surgery at an outside hospital revealed a 3.5 × 2.5 × 2.5 cm myxoma consisting of a complex papillary structure comprising grape-like clusters organized into an arborizing network (Fig. 2a–g). Microscopy of histologic sections prepared at Yale from two paraffin tissue blocks confirmed the myxoma’s arborizing structure lined by lepidic cells overlying the majority of the surfaces. The tumor’s myxoid matrix was interspersed with clusters and linear strings of cells comprising abortive endothelial-like structures (Fig. 2c–e). In addition, at the surface there were several foci of clusters of rounded, hyperchromatic cells displaying a lack of cohesion, forming small collections of cells separating from the surface of the myxoma (Fig. 2f, g).

Tumor tissue obtained from the brain biopsy in an imaging-confirmed area at Yale New Haven-Smilow Hospital was studied by microscopy and immunohistochemistry. Staining with an anti-calretinin antibody revealed an arterial vessel with its endothelial lining replaced by calretinin-positive myxoma cells that had infiltrated focally into adjacent brain parenchyma (Fig. 3a, b). The subendothelium of the vessel was replaced with myxoid matrix and myxoma cells that were seen invading through the media, breaching the internal and external elastic lamina (dashed lines; Fig. 3b, c). The myxoma tumor cells also stained positively for SMA and CD31 (data not shown).

In 1997 our patient underwent bilateral ovarian biopsies at an outside hospital, from which the diagnosis of papillary serous cystadenomas was made. The slides were reviewed at the time of her current admission and the diagnosis confirmed. Sections cut from the requested paraffin blocks of the cardiac myxoma and the ovarian tissues from the outside hospitals were utilized for whole exome sequencing.

## Whole exome sequencing

### Methods

Deoxyribonucleic acid (DNA) was extracted from tumor tissue microdissected from sections after deparaffinization. The concentration of tumor cells in the regions dissected was estimated from adjacent sections cut from the paraffin blocks. DNA from buccal swabs (Isohelix) was prepared by methods similar to those used for tissue, except for skipping deparaffinization and reversal of formalin crosslinking. The DNA was resuspended in a buffer solution (10 mM Tris, 0.1 mM EDTA) and DNA concentration determined using the Qubit® 2.0 Fluorometer (Thermo Fisher Scientific).

Libraries of DNA fragments for sequencing were prepared by multiplex polymerase chain reaction (PCR) of the genomic DNA. The Ion AmpliSeq™ Exome RDY 4×2 panel (Thermo Fisher Scientific) was used for whole exome sequencing of the heart valve, left ovary, right ovary, and brain samples. The amounts of input genomic DNA for whole exome sequencing were 80 ng for the heart valve, 100 ng for the brain, and 60 ng for the ovarian sample. The Ion AmpliSeq Comprehensive Cancer Panel (Thermo Fisher Scientific) (primer pools 2 and 3 only) was used to prepare libraries for targeted DNA sequencing of the heart valve and right ovary (10 ng input DNA for each pool).

The PCR, amplicon digestion, and barcode adapter ligation were performed using Eppendorf® Mastercycler® Pro S Thermal Cyclers (Eppendorf). Libraries were quantitated by quantitative PCR (qPCR) using the Ion Library TaqMan® Quantitation Kit (Thermo Fisher Scientific) and the ViiA 7 real-time PCR system (Thermo Fisher Scientific). DNA libraries from tumor and buccal swab germline DNA were separately barcoded, mixed in a 3:1 tumor to germline ratio, and sequenced together.

Sequences obtained for fragments were aligned to the hg19 reference sequence using the Ion Torrent Suite™ software version 5.2 (Thermo Fisher Scientific). Variant calling and annotation were performed by the Ion Reporter™ software version 5.0 (Thermo Fisher Scientific) using the AmpliSeq™ Exome tumor-normal pair workflow and the AmpliSeq™ CCP single sample workflow for whole exome and targeted sequencing, respectively. Called variants were individually inspected and evaluated using the Integrative Genomics Viewer (IGV).

### Results

The microdissected tissue from the heart was estimated to contain approximately 90% tumor cells. The whole exome sequencing of DNA from this lesion had a mean read depth of 223.3 nucleotides, along with a mean read depth of 171.8 for the accompanying germline DNA. The assembled sequence showed 46 somatic mutations – variants not found in the germline DNA collected by buccal swab – within or closely bordering the coding sequences of 39 different genes. Attention was focused on 12 mutations that were non-synonymous, and had a variant allelic fraction (VAF) of ≥ 10 with a read depth of > 50×. Four of these mutations involved the *PRKAR1A* gene (one missense mutation, one insertion, one deletion, and a 5× amplification; Table 1). In addition, a synonymous mutation was found within *PRKAR1A*. None of the other mutations were in genes that have been generally implicated in tumor cell proliferation or survival.

**Table 1:**
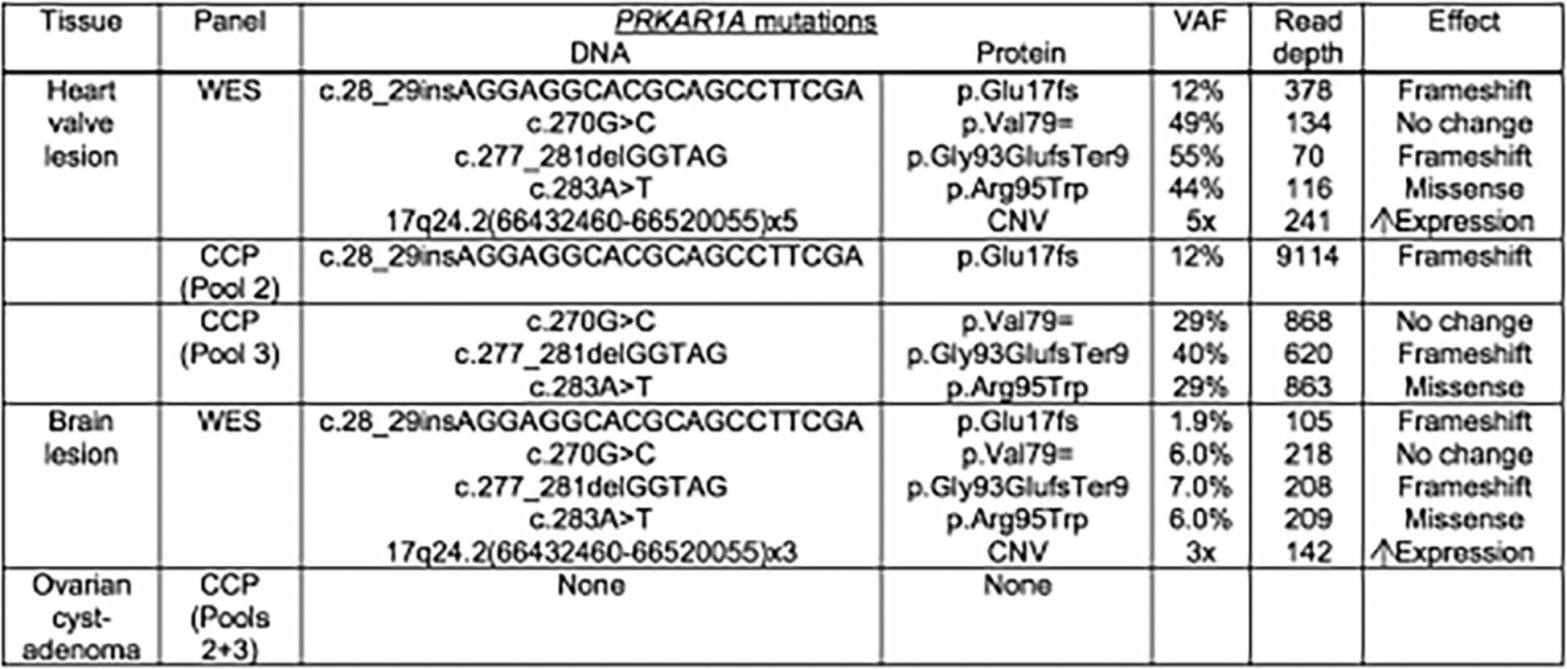
*PRKAR1A* mutations detected within the tissues

*CCP* Ion AmpliSeq™ Comprehensive Cancer Panel, *CNV* copy number variation, *VAF* variant allelic fraction, *WES* whole exome sequencing. Read depth is the total number of reads covering the site of the mutation, except for the amplification, for which read depth refers to the mean read depth across the entire amplified region. The mean read depth for the amplified region in normal DNA was 107. The amplified region in both the heart and brain lesions contains *PRKAR1A* and the gene *WIPI1*

Three of the mutations in *PRKAR1A* (the missense and synonymous mutations, and a five-base pair deletion) were tightly clustered within the gene and were present at comparable VAFs (44–55%). The phase of a 20-base pair insertion present at 12% could not be directly determined with respect to these three mutations based on next generation sequence analysis, in which DNA is sequenced in small fragments (average read length 175 nucleotides).

To confirm the presence of the *PRKAR1A* mutations and better assess the VAFs for these alterations, targeted sequencing was carried out on the same lesional DNA using pools of multiplex primers from the Ion AmpliSeq™ Comprehensive Cancer Panel that separately covered the regions of the mutations within the gene. The mutations detected by whole exome sequencing were again found by targeted sequence with roughly the same VAF score for the five-base pair deletion and at a slightly reduced level for the missense mutation (VAF 41%, 250/617 reads and 29%, 249/863 reads, respectively). The 20-base pair insertion was found at an identical VAF (12%, 1027/8409 reads). Resequencing of DNA from normal tissue using the Comprehensive Cancer Panel failed to detect any of the *PRKAR1A* mutations, with an average read depth of 1852 covering the regions containing those mutations.

The lesional tissue examined from the brain had a much lower concentration of tumor cells, estimated to be approximately 20%. All of the *PRKAR1A* mutations identified in the heart tissue were present in the brain tissue, albeit at much lower VAFs (the 5-base pair deletion at 7%, the missense and synonymous mutations at 6% each, and the 20-base pair insertion at 1.9%).

Mutations within the *PRKAR1A* gene were not detected in DNA from the cystadenoma of the right ovary using the Comprehensive Cancer Panel. Attempts to sequence DNA extracted from the much less abundant tissue available from the cystadenoma of the left ovary yielded results that did not meet quality metrics*.*

## Discussion and conclusions

In general, atrial myxomas are pathologically benign lesions; however, their malignant potential has been described in small case series and individual case reports in the literature [4, 9, 15–25]. The CNS is the most common location for remote growth of myxomatous material. Autopsy series and retrospective reviews of patient cohorts have estimated the incidence of cerebral parenchymal metastases to be between 1.8 and 4.5% of patients with myxomas [12, 15]. Risk factors leading to metastasis are poorly understood and the clinical and pathological features of patients with cerebral parenchymal metastases are varied. In several case reports of myxoma metastases, metastatic lesions appeared more cellular and pleomorphic than the original tumor, suggesting there had been malignant transformation [26]. In another report of a patient with sarcomatous-appearing metastases and a history of benign cardiac myxoma, a retrospective review of the initial cardiac tissue revealed an area at the periphery of the tumor that had more malignant features, suggesting a possible “malignant myxoma” at the outset [15]. Other patients had metastatic disease at distant sites from typical benign-appearing myxomas without any aggressive features on histopathology [16, 21].

As demonstrated in our case, myxoma cells from tumor emboli to the CNS vasculature can attach to the endothelial wall, weaken the endothelium, and invade the elastic lamina leading to vascular wall dilatation and so called “oncotic-aneurysm” formation [27]. The natural history of these aneurysms is not certain given their rarity and varied outcomes. Lesions have been reported to behave anywhere on the spectrum from self-resolving to enlarging and may cause both ischemic and hemorrhagic events [28, 29]. In rare circumstances, as evidenced by our patient’s pathology, myxoma cells may penetrate through the vessel wall of these aneurysms and into the surrounding brain tissue, leading to the formation of parenchymal lesions. This mechanism has been demonstrated previously and other case reports of brain metastases describe cerebral aneurysms on imaging in association with parenchymal lesions [12, 30]. However, not all reported cases of brain metastases describe these imaging findings. Factors affecting the ability of tumor cells to disseminate to the brain, penetrate blood vessel walls, and proliferate within the brain remain to be identified. Prior work has suggested that production of IL-10 by the tumor may play a role, but further studies are needed [25, 31].

Genetic analysis of our patient’s tissue showed multiple mutations within the *PRKAR1A* gene in both the brain metastasis and cardiac tumor sample, but not within tissue derived from a benign ovarian cystadenoma or a buccal DNA swab. While ovarian cystadenomas may be a feature of Carney complex, the absence of *PRKAR1A* mutations in the cystadenoma suggests that her heart tumor was a sporadic myxoma. The failure to find the *PRKAR1A* mutations in the material collected by buccal swab is consistent with this conclusion, although there is a very small possibility that our patient is mosaic for a mutation at a very low level of mutant cells and that the buccal mucosa and ovarian tissues were spared the mutation present in other somatic tissues. The *PRKAR1A* gene encodes the type 1A regulatory subunit of the cyclic adenosine monophosphate (cAMP)-dependent enzyme protein kinase A (PKA) and functions as a tumor suppressor [32]. The PRKAR1A protein exerts a repressive effect on the kinase activities of the PKA complex. Mutations inactivating this subunit lead to increased cell proliferation in cAMP-responsive tissues and are believed to play a role in tumorigenesis. All of the intragenic mutations detected within the *PRKAR1A* gene in this case (with the exception of the synonymous mutation) should inactivate the protein encoded by the allele containing the mutation. Both indel mutations result in frameshifts and the missense mutation is predicted to have a deleterious effect on the structure and function of the protein, according to the SIFT and PolyPhen prediction programs. Whether both alleles are affected by these mutations is not possible to determine by next generation sequencing, so the extent to which *PRKAR1A* activity is lost within the tumor cells is not known. However, the 5× amplification (five copies of the *PRKAR1A* gene) suggests that the allele carrying the deletion, missense, and synonymous mutations is the allele that underwent amplification, resulting in four copies of that allele within the tumor cells of the heart. This ratio was somewhat lower (3×) in the metastatic tumor analyzed from the brain. If this interpretation is true, both *PRKAR1A* alleles had suffered deleterious mutations. (The amplification of the tumor suppressor is unexpected and unexplained. Amplification is usually associated with oncogenes, not tumor suppressors like *PRKAR1A*, and may coexist with activating mutations within the amplified genes).

The VAFs for the mutations in genes other than *PRKAR1A* found in the tumor cells (not shown) – all approximately 25% – are consistent with a tumor cell concentration of approximately 50% in the tissue analyzed (despite a higher estimate by histopathology), assuming that these mutations are heterozygous. That the insertion mutation in PRKAR1A is lower (12%) probably reflects the likely amplification in the opposite allele. The absence of detectable PRKAR1A mutations in the germline DNA from the buccal swab and right cystadenoma of the ovary argues against mosaicism for Carney complex, although ovarian cystadenomas have been noted to be more prevalent in patients with Carney complex compared to the general population. In this case, the ovarian cystadenomas resected many years earlier were probably sporadic and coincidental with the later development of a cardiac myxoma.

There has been debate as to whether mutations in *PRKAR1A* play a role in the development of isolated cardiac myxoma. Initially, genetic analyses conducted on small patient cohorts with isolated cardiac myxomas showed that mutations were not found in these cases. In two recent studies, *PRKAR1A* mutations were found in sporadic cardiac myxomas. In one of these studies, 33 out of 103 tumors lacked expression of the PRKAR1A protein and nine patients had mutations in *PRKAR1A* identified [7]. A subsequent study found that seven out of eight sporadic tumors had mutations in *PRKAR1A* on whole exome sequencing analysis [8]. Whether this mutation alters the clinical course of sporadic myxomas is not clear. On histopathologic examination, myxomatous tissues from hereditary and isolated myxomas appear very similar. Although a cardiac myxoma associated with Carney complex is more likely to recur, there are no data to the best of our knowledge that these tumors are more likely to metastasize. In addition, prior case reports on brain metastases have not included whole exome sequencing data from the tumor tissues.Unfortunately, our patient’s lesions grew quickly and caused significant neurological deficits, prompting the rapid initiation of treatment. As brain metastases are an infrequent complication of a rare tumor, there is no standard accepted treatment and therapeutic strategies derive from experience with small numbers of patients. There are as yet no available agents to target the *PRKAR1A* mutation. Therapy with doxorubicin has been attempted in a patient with an oncotic aneurysm but the aneurysm continued to enlarge despite treatment [28]. Altundag *et al.* treated a patient similar to ours with whole brain radiotherapy and achieved stability for at least 4 years (the patient was still alive and stable at time of publication) [16]. Another report described decrease in size of multiple parenchymal lesions with whole brain radiotherapy [33]. A third patient was treated with whole brain radiotherapy followed by chemotherapy with ifosfamide and doxorubicin and achieved long-term control [34]. Our patient’s lesions showed partial response to whole brain radiotherapy and she has now been stable by imaging and clinical criteria for 1 year.

Our case illustrates a rare occurrence of atrial myxoma with brain parenchymal metastases and highlights the ability of myxoma cells to migrate though cerebral vessel walls, form an oncotic aneurysm, and then invade the brain parenchyma. It also provides further evidence that *PRKAR1A* mutation can occur in sporadic myxomas. The precise molecular features and risk factors that allow myxoma cells to invade surrounding tissue have yet to be elucidated. Among genes recognized as having functions related to tissue invasion and metastasis, none were found to be mutated within the tissue of the heart or brain lesions. Whether the *PRKAR1A* mutation in sporadic myxomas alters the clinical course of disease or increases the likelihood of metastasis is not clear. Further information on the genotype–phenotype correlation of *PRKAR1A* mutations is needed to help predict the clinical behavior of tumors with this abnormality. In addition, the PKA pathway regulated by *PRKAR1A* offers a possible target for systemic therapy for tumors harboring *PRKAR1A* mutations, in particular those tumors associated with the most serious complication of cardiac myxomas: cerebral metastases.

## Data Availability

We will submit the sequence data to a database following acceptance.

## Abbreviations

cAMP: Cyclic adenosine monophosphate
CNS: Central nervous system
CT: Computed tomography
FLAIR: Fluid-attenuated inversion recovery
GRE: Gradient recalled echo
ICA: Internal carotid artery
PCR: Polymerase chain reaction
PKA: Protein kinase A
SWI: Susceptibility weighted imaging
TTE: Transthoracic echocardiogram
VAF: Variant allelic fraction
